# The Significance of CXCL1 in Cancer: An Overview of Molecular Mechanisms

**DOI:** 10.3390/ijms27062693

**Published:** 2026-03-16

**Authors:** Jan Korbecki, Mateusz Bosiacki, Edyta Dzięciołowska-Baran, Patrycja Pawlik, Michał Lubkowski, Ireneusz Walaszek, Katarzyna Barczak

**Affiliations:** 1 Institute of Health Sciences, Collegium Medicum, University of Zielona Góra, Zyty 28 St., 65-046 Zielona Góra, Poland; 2 Department of Biochemistry and Medical Chemistry, Pomeranian Medical University in Szczecin, Powstańców Wlkp. 72 St., 70-111 Szczecin, Poland; mateusz.bosiacki@pum.edu.pl; 3 Department of Anatomy, Pomeranian Medical University in Szczecin, Powstańców Wlkp. 72 St., 70-111 Szczecin, Poland; edyta.dzieciolowska.baran@pum.edu.pl; 4 Department of Endodontic Surgery, Pomeranian Medical University in Szczecin, Powstańców Wlkp. 72 St., 70-111 Szczecin, Poland; patrycja.pawlik@pum.edu.pl (P.P.); katarzyna.barczak@pum.edu.pl (K.B.); 5 Department of Medical Biology, Pomeranian Medical University in Szczecin, Powstańców Wlkp. 72 St., 70-111 Szczecin, Poland; michal.lubkowski@pum.edu.pl; 6 Department of Nursing, Pomeranian Medical University in Szczecin, Żołnierska 48 St., 71-210 Szczecin, Poland; ireneusz.walaszek@pum.edu.pl

**Keywords:** CXCL1, CXCR2, tumor, cancer, neutrophil, inflammation, chemokine

## Abstract

Chemokine CXCL1, also known as Gro-α and MGSA, a ligand of CXCR2, is the best-known CXC chemokine in cancer processes, after CXCL8/IL-8 and CXCL12/SDF-1. This paper is the first review on the role of CXCL1 in general molecular processes associated with cancer. It provides a comprehensive overview that allows for an in-depth understanding of the importance of CXCL1 in tumor-related processes. In this review, however, we did not address the clinical aspects of CXCL1, as these were discussed in our previous review articles. The present paper focuses on the involvement of CXCL1 in cancer processes such as proliferation, cancer stem cell (CSC) function, senescence, angiogenesis, lymphangiogenesis, migration and metastasis, and effects on tumor-associated cells such as neutrophils, tumor-associated macrophages (TAMs), myeloid-derived suppressor cells (MDSCs), mesenchymal stem cells (MSCs), and cancer-associated fibroblasts (CAFs). It also describes the significance of CXCL1 in cancer-associated diseases such as cancer cachexia, cancer-associated immunodeficiency, neuroinflammatory-mediated affective-like behaviors, bone cancer pain, and acute kidney injury. We also present the effects of obesity on CXCL1-related cancer processes.

## 1. Introduction

Chemokines are cytokines that cause chemotaxis of immune cells [1]. Forty-six different chemokines identified in humans are divided into sub-families depending on the cysteine motif at their N-terminus: α-chemokines with a CXC motif; β-chemokines with a CC motif; γ-chemokines with an XC motif; and δ-chemokines with a CX_3_C motif. CXC chemokines are numbered from 1 to 17 [1,2]. In humans, there is also a non-allelic CXC motif chemokine ligand 4 variant-1 (CXCL4L1) for the CXC motif chemokine ligand (CXCL)4 chemokine [3]. While CXCL15/lungkine can be found in rodents, in humans, it only occurs as a pseudogene in the 4q12-q13 CXC chemokine gene cluster alongside the genes of CXC motif chemokine receptor (CXCR)2 ligands and chemokine platelet-derived factor 4 (PF4)/CXCL4 [4]. The CXC chemokine sub-family can be further divided according to the receptor that is activated. The chemokines CXCL1-8 (except PF4/CXCL4) activate CXCR2 [1,2]. PF4/CXCL4 and CXCL9-11 activate CXCR3. CXCL12, CXCL13, CXCL16, and CXCL17 activate CXCR4, CXCR5, CXCR6, and G-protein-coupled receptor 35 (GPR35)/CXCR8, respectively. In humans, there are seven CXCR2 ligands [1,4], with CXCL6 and CXCL8 also activating CXCR1 at the same concentration as CXCR2 [5,6,7]. Other CXCR2 ligands also activate CXCR1 but not until concentrations are about 100 times higher [5,6,7]. In this way, the biological properties of these chemokines are associated with the activation of CXCR2. Isolated studies show that CXCL1 action depends on CXCR1 and CXCR2 [8]. The third receptor for CXCL1 is atypical chemokine receptor 1 (ACKR1)/Duffy antigen receptor for chemokines (DARC) [1,9]. This receptor can only regulate CXCL1 levels [10]. However, ACKR1/DARC can inhibit CXCR2 activity [11]. If it is located on the same cancer cell as CXCR2, then ACKR1/DARC has an anti-tumor effect. This paper is the first to summarize the body of research on the role of CXCL1 in the development of many cancers.

## 2. Research Method

This review is based on experimental articles on CXCL1 in cancer indexed in the PubMed search engine (https://pubmed.ncbi.nlm.nih.gov, accessed on 9 October 2025). Articles were filtered by the phrase: (MGSA or CXCL1 or GROA or GRO-A or GRO-1 or GRO1) cancer, not review. The main phrase “MGSA or CXCL1 or GROA or GRO-A or GRO-1 or GRO1” contains the historical and current names of the CXCL1 chemokine with Greek letters converted to Latin. It was used to find all available articles that had the name of the chemokine in the title, abstract, and keywords. The word “cancer” narrowed the search to articles on the role of CXCL1 in cancer. The phrase “not review” excluded review papers from the search. Out of the initial search result of 1200 articles, about 600 articles were pre-selected for reading based on the title. We also used their references to find any additional articles that could be relevant to this review.

One problem in writing this review was the lack of an animal model of CXCL1 action [4,11]. In humans, there are seven CXCR2 ligands, while in mice, there are five. The common ancestor of humans and mice had few CXCR2 ligand genes. The current genes in humans and mice appeared independently through duplication and speciation, and in each species, each CXCR2 ligand acquired its own characteristic properties. In addition, because a variety of processes are associated with the simultaneous expression of many different CXCR2 ligands, it is impossible to find the exact equivalents of individual human CXCR2 ligands in mice. For this reason, when discussing the articles demonstrating the importance of murine “CXCL1” in a given process, we preferred to be more general and write that a given cancer process only increases the expression of CXCR2 ligands in a mouse model. For this reason, studies conducted in mouse models serve mainly to confirm findings obtained in vitro using human cancer cell lines and analyses of human tumor tissues. Nevertheless, in this review, we attempt to avoid citing experimental studies performed in mouse models whenever possible.

## 3. Regulation of CXCL1 Expression in Cancer Cells

CXCL1 is produced by cancer cells, as demonstrated by analyses of models such as bladder cancer [12], cervical cancer [13], colorectal cancer—particularly with mutations in the *KRAS* gene [14]—gastric cancer [15,16], melanoma [17,18], prostate cancer [19], and many others.

Increased expression of CXCL1 in a cancer cell may be the result of the genetic changes that underlie the development of tumors. Frequent gene amplification leads to an increase in the expression of these genes, although the frequency of this amplification of the *CXCL1* gene is low and depends on the type of cancer. It occurs in about 2.5% of ovarian cancer cases [20,21], 2% of breast cancer cases [22], and 1% of head and neck squamous cell carcinoma cases [21]. Deletions or missense mutations in the *CXCL1* gene also occur, but they are fewer than amplifications and involve only isolated cases [23,24,25]. The frequency of CXCL1 amplification increases with tumor growth. For this reason, a higher frequency of *CXCL1* gene amplification is observed in metastasis, as shown by studies on breast cancer [26]. Genetic changes associated with CXCL1 may also contribute to cancer predisposition. An example of this is the duplicated region on chromosome 4q13 [27]. This is the region with the locus of the *CXCL1* gene and the genes of other CXCR2 ligands.

An increase in CXCL1 expression in a tumor can result from genetic changes in other genes. An example of this is mutations in *TP53* causing an increase in CXCL1 expression. *TP53* is a tumor suppressor gene. If the product of this gene, p53, is activated, it inhibits proliferation and causes apoptosis upon stress induced by damage in the cell as well as by DNA damage and accumulation of mutations [28]. On the other hand, mutations in this gene are common in cancer [29]. The reason for this is selection pressure over cells with a reduced capacity for apoptosis [30]. *TP53* mutations can be divided into loss-of-function and gain-of-function mutations. A loss-of-function *TP53* mutation, such as in the DNA-binding domain, leads to increased CXCL1 expression [31]. Nuclear factor κB (NF-κB) and p53 mutually inhibit each other’s activity [32]—for this reason, a loss-of-function *TP53* mutation leads to increased NF-κB activity and expression of genes dependent on this transcription factor, including CXCL1. Also, a gain-of-function mutation of *TP53, for example,* in the Zn^2+^-binding domain, results in an increase in CXCL1 expression [31,33]. This is associated with an increase in H-Ras activity [31]. p53 with this mutation attaches to the promoter of the *CXCL1* gene [33], resulting in a direct increase in the expression of CXCL1 in a cancer cell with this *TP53* mutation. Also, DNA-contact p53 gain-of-function mutants attach to the *CXCL1* promoter, thus increasing the expression of this gene [31,34].

Another protein belonging to the p53 transcription factor family is p63 [35]. Members of this family increase the expression of similar genes but can also differ in their effects on the expression of certain genes. p63 attaches to the *CXCL1* promoter to increase the expression of this gene [36]. This process is significant in the development of pancreatic ductal adenocarcinoma.

CXCL1 expression in a cancer cell is dependent on high basal NF-κB activation [18,37,38]. Various factors are responsible for this feature of the tumor cell, including the elevated expression of NF-κB-inducing kinase (NIK) [39], mutation in the *TP53* gene and non-functionality of p53 [40], and elevation in tumor levels of pro-inflammatory cytokines such as tumor necrosis factor-α (TNF-α) [41] and interleukin (IL)-1α and IL-1β [42,43]. NF-κB attaches to the promoter of the *CXCL1* gene, which increases the expression of this chemokine. At the same time, once secreted outside the tumor cell, CXCL1 activates its receptor CXCR2 in an autocrine manner. Subsequently, CXCL1 increases the activation of NF-κB. Activation of this transcription factor, which depends on p38 mitogen-activated protein kinase (MAPK) but not extracellular signal-regulated kinase (ERK) MAPK activation [44], leads to an increase in CXCL1 production in the tumor cell by CXCL1 itself. This process has been demonstrated in cervical cancer [13], malignant melanoma [17,44,45], and prostate cancer [19].

Other transcription factors can increase CXCL1 expression in a tumor cell. An example of this is Snail [46], a transcription factor responsible for epithelial-to-mesenchymal transition (EMT) [47]. Snail increases CXCL1 expression by directly attaching to the CXCL1 promoter and by increasing NF-κB activation [46]. For this reason, migrating tumor cells express CXCL1, which is important in tumor growth, invasion and metastasis. Another protein that attaches to the CXCL1 promoter is hes-related family bHLH transcription factor with YRPW motif like (HEYL) [48], activated by Notch signaling, a signaling pathway frequently activated in tumors [49]. This mechanism of increased CXCL1 expression is important in the induction of angiogenesis via Notch signaling.

CXCL1 expression can also be regulated by histone modification. Specifically, CXCL1 expression can be reduced by histone H3 lysine 36 methyltransferase SET-domain-containing 2 (SETD2) [50]. SETD2 induces histone methylation at positions ranging from 2.0 to 1.5 k base pairs prior to the onset of transcription at the start of the *CXCL1* gene, leading to the decreased expression of this chemokine. Importantly, mutations in the *SETD2* gene and decreased SETD2 expression are common in various cancers [50,51,52] and lead to increased CXCL1 expression.

High expression of CXCL1 in a cancer cell may also be due to the increased activity of RAS proteins, particularly K-RAS. In tumors, mutations in RAS genes often occur, causing an increase in the activity of these proteins. An increase in CXCL1 expression caused by mutation in RAS protein genes was confirmed by experiments on colorectal cancer cells [53] and pancreatic ductal adenocarcinoma [54,55]. This process is important in the emergence of autocrine cell growth and the development of many cancers.

CXCL1 expression in the tumor may depend on factors present in the tumor microenvironment. It may depend on chronic hepatitis B virus (HBV) and hepatitis C virus (HCV) infection in liver cancer [56,57,58,59], *Helicobacter pylori* infection in gastric cancer [60,61], Kaposi’s sarcoma-associated herpesvirus (KSHV)/human herpes virus-8 (HHV8) and human immunodeficiency virus (HIV) infection in Kaposi’s sarcoma [62]. Also, CXCL1 expression in the tumor cell is increased by prostaglandin E_2_ (PGE_2_) as well as other prostanoids [63,64,65]. At the same time, in colorectal cancer, this may be the most important factor responsible for the elevated CXCL1 expression in the tumor [65]. CXCL1 expression is also increased by secretory factors such as CXCL12/stromal-derived factor-1 (SDF-1) [66], IL-1α and IL-1β [42,43], epidermal growth factor receptor (EGFR) ligands [67], and IL-17 [68].

Not all factors increase CXCL1 expression–some decrease it. An example of this is the transforming growth factor β (TGF-β), which decreases CXCL1 expression [69]. Therefore, the disruption of TGF-β increases the expression of CXCL1. The disruption of SMAD family member 4 (SMAD4) [70] or the loss of TGF-β type II receptor (TβRII) expression in cancer-associated fibroblasts (CAFs) is common in tumors [71]. This leads to increased CXCL1 expression.

CXCL1 expression is regulated by microRNA. At the same time, the expression of microRNA in cancer is often downregulated relative to healthy tissue. This leads to an increase in CXCL1 expression. Examples include miR-1-3p in rectal adenocarcinoma [72], miR-27b-5p in ovarian cancer [73], miR-141 in non-small-cell lung cancer [74], miR-145-5p in colorectal cancer [75], miR-200a in hepatocellular carcinoma [76,77], and miR-302e in colorectal cancer [78].

CXCL1 expression may also depend on changes in the tumor microenvironment, such as hypoxia, i.e., a reduction in oxygen levels in certain regions of the tumor. In tumors, there are regions characterized by chronic hypoxia and cycling hypoxia [79,80]; the latter exhibits hypoxia, and the reoxygenation of such areas occurs from time to time. Hypoxia affects CXCL1 expression, but this is dependent on the model studied. In a model of hepatocellular carcinoma cells [81], cervical cancer cells [82], and prostate cancer cells [83], chronic hypoxia increases CXCL1 expression. Conversely, in SK-OV-3 ovarian adenocarcinoma and WM793B melanoma cells, chronic hypoxia reduces CXCL1 expression [83]. In contrast, in lung adenocarcinoma cells, chronic hypoxia does not affect CXCL1 expression [84]. Cycling hypoxia, depending on the model, affects CXCL1 expression in various ways. It can increase expression in PC-3 prostate cancer cells and SK-OV-3 ovarian adenocarcinoma cells and also decrease the expression of CXCL1 in WM793B melanoma cells [83]. Another parameter of the tumor microenvironment is reduced pH or acidosis [85], which increases CXCL1 expression, as shown by experiments on bone marrow mesenchymal stem cells (MSCs) [86]. CXCL1 expression in a tumor may depend on tumorigenic changes in extracellular matrix (ECM). Unfolding of the type III domains of fibronectin is caused by myofibroblasts [87]. Such an altered ECM protein causes an increase in the expression of CXCR2 ligands, including CXCL1 and TNF-α, in fibroblasts. This process has been noted in lung squamous cell carcinoma tumors [87].

The action of CXCL1 can be dependent on CXCR2 and on its other receptors, for example, ACKR1/DARC [9], an atypical receptor for CXCL1 and other chemokines. This receptor binds this chemokine without clear intracellular signal transduction. Therefore, ACKR1/DARC regulates the availability of CXCL1 to the CXCR2 receptor by removing this chemokine from the extracellular space [9]. Also, if the expression of ACKR1/DARC occurs on the same cell as CXCR2, then it can interfere with CXCR2 function [11,88].

Cell death is associated with the formation of residues that should be removed by phagocytic cells. For this reason, during cell death, there is an increase in the secretion of chemotactic factors for neutrophils and monocytes. This is the so-called “find-me” signal. One of them is CXCL1. The expression of this chemokine is increased during FasL-induced apoptosis [89], which is associated with NF-κB activation by the Fas-associated via receptor interacting serine/threonine kinase 1 (RIPK1) complex.

An increase in CXCL1 expression also occurs during necroptosis [90,91], a process that has features of apoptosis and necrosis. It is a process of programmed cell death that includes the violation of cell membrane integrity. Increased expression of RIPK1 and RIPK3, the components of necroptosis, occurs during chemotherapy, for example, when using gemcitabine against pancreatic ductal adenocarcinoma cells [90]. This increases CXCL1 expression in the tumor after chemotherapy, which is important in resistance to chemotherapy. This process is common in many types of cancer treated with many types of anticancer drugs. For example, CXCL1 expression was found to be increased in tumor cells treated with chemotherapeutics such as paclitaxel [92,93], oxaliplatin [94,95], and epidoxorubicin [96]. Long-term reduction in RIPK3 expression can increase CXCL1 expression through increased NF-κB activation, as noted in hepatocellular carcinoma cells [97].

## 4. Molecular Mechanism of CXCL1 Function in Cancer Processes

### 4.1. Proliferation and Pro-Survival Properties

CXCL1 increases tumor cell proliferation in many types of cancer, including bladder cancer [98], cervical cancer [13], colorectal cancer [99], gastric cancer [100,101], glioblastoma [102], and ovarian cancer [103]. However, this property depends on the type of cancer—for example, CXCL1 weakly increases prostate cancer cell proliferation [8], while in cholangiocarcinoma, CXCL1 inhibits tumor cell proliferation [104].

**Figure 1:**
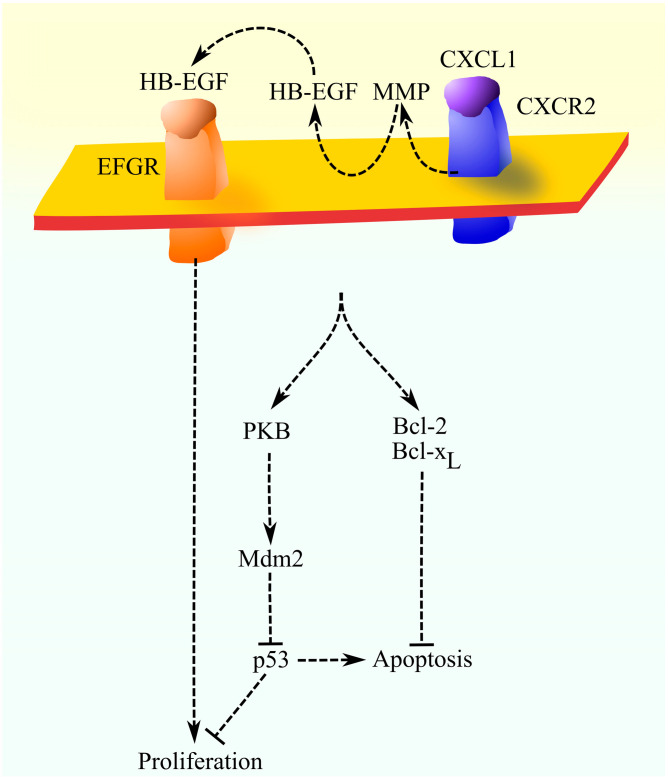
The role of CXCL1 in cancer cell proliferation. CXCL1 activation of CXCR2 triggers the transactivation of EGFR, a process mediated by increased MMP activity, which releases HB-EGF. This growth factor subsequently activates EGFR, promoting cancer cell proliferation. Additionally, CXCL1 exerts anti-apoptotic effects by upregulating Bcl-2 and Bcl-x_L_, the key proteins that inhibit apoptosis. Through CXCR2 signaling, CXCL1 also activates the Akt/protein kinase B (PKB)–murine double minute 2 (Mdm2) pathway, leading to the suppression of p53 activity. This reduction in p53 function removes its inhibitory effects on cell proliferation and apoptosis induction, further contributing to tumor growth.

Increased proliferation by CXCL1 is associated with the activation of CXCR2, which causes the transactivation of EGFR (Figure 1) [105,106]. Activation of CXCR2 causes signal transduction and activation of an unspecified matrix metalloproteinase (MMP), as indicated by the used pan-MMP inhibitor GM6001 [105]. Activated MMP causes the proteolytic cleavage of heparin-binding EGF-like growth factor (HB-EGF), a ligand of EGFR. HB-EGF then activates its receptor EGFR.

The increase in tumor cell proliferation by CXCL1 occurs because of CXCR2 activation, which leads to decreased expression of p21, a cell cycle inhibitor at the G_1_/S checkpoint [107]. CXCR2 also causes an increase in the expression of cyclins and cyclin-dependent kinases, including cyclin A, cyclin B1, cyclin D1, cyclin E, cyclin-dependent kinase (CDK)2, and CDK6 [107]. Importantly, the effect on proliferation depends on the status of p53 in the cancer cell [108]. CXCR2 activation increases the proliferation of cells with p53wt much more than that of cells with either p53 mutation or non-functional p53. This is related to the activation of the Akt//PKB-Mdm2 pathway. Mdm2 inhibits p53 activity, which leads to reduced expression of p21, a cell cycle inhibitor [108].

CXCR2 activation has an anti-apoptotic effect [107,109]. It is associated with an increase in the expression of anti-apoptotic Bcl-2 family proteins, i.e., Bcl-2 and Bcl-x_L_, a decrease in the expression of pro-apoptotic Bcl-2 family proteins, and the inhibition of p53 activity.

The action of CXCL1 is partly autocrine [13]. The cancer cell has high basal activation of NF-κB [37,38], resulting in high expression of genes dependent on this transcription factor, such as CXCL1. Subsequently, through its receptor, CXCL1 increases NF-κB activation and thus its own expression [44].

The action of CXCL1 may also depend on the interaction of various cells in the tumor niche. A significant source of CXCL1 in some cancers is CAF [110]. The tumor cell secretes factors that turn fibroblasts into CAFs; CAFs then secrete CXCL1, which increases tumor cell proliferation.

Another aspect of CXCL1’s influence on cancer cells is its effect on metabolism. A study on colorectal cancer has shown that CXCL1 increases the intensity of glycolysis by increasing the expression of glucose transporter type 1 (GLUT1), hexokinase 2 (HK2), and lactate dehydrogenase A (LDHA) [111]. As a consequence, cancer cells secrete lactate, which shapes the tumor microenvironment and influences multiple cancer-related processes.

### 4.2. Senescence

In an unfavorable environment that causes damage to DNA, and due to the accumulation of genetic changes over the years [112], a cell can be transformed into a non-dividing senescent cell to prevent uncontrolled proliferation from the mutation of genes encoding RAS proteins [113,114].

**Figure 2:**
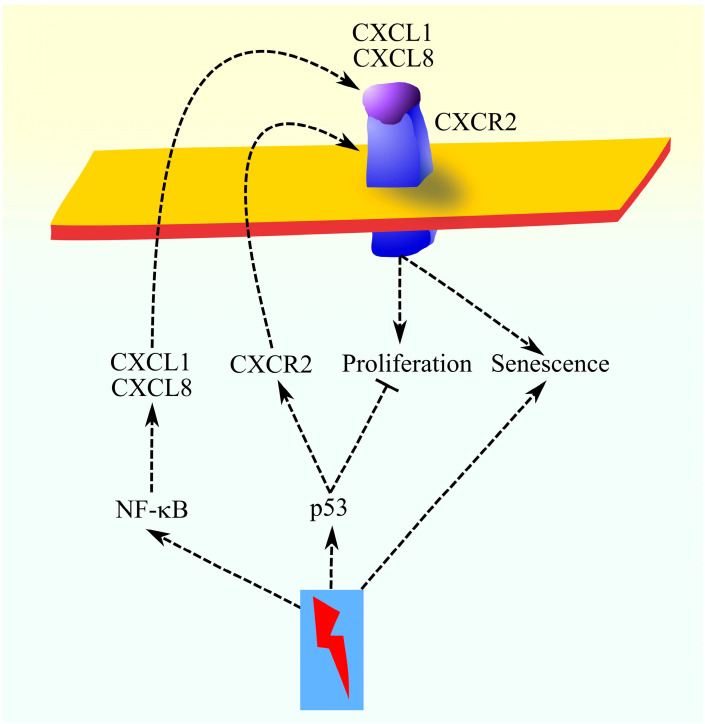
CXCL1 in cellular senescence. Stress-induced cellular senescence is driven by the activation of NF-κB and p53. NF-κB upregulates the expression of CXCL1 and CXCL8, while p53 increases the expression of their receptor, CXCR2, leading to receptor activation. CXCR2 signaling reinforces and maintains the senescent state of the cell but does not promote proliferation due to p53-mediated inhibition. However, if p53 function is compromised, CXCR2 activation can drive cell proliferation, potentially leading to oncogenic transformation of previously senescent cells.

Cellular senescence involves p53-dependent inhibition of cell division [115]. In addition, the cell shows changes in the secretion of a variety of factors, the so-called senescence-associated secretory phenotype (SASP) [115,116], causing the migration of immune cells that remove the pre-malignant senescent cells [117]. The SASP induction is dependent on NF-κB [113,118], which causes an increase in the expression of CXCL1 and CXCL8/IL-8 [113,116]. At the same time, activated p53 binds to the CXCR2 promoter that elevates the expression of this receptor in senescent cells and results in enhancements of the action of ligands of this receptor, such as CXCL1 and CXCL8/IL-8 [114]. These two chemokines cause an increase in the expression of CXCL1 and CXCL8/IL-8, as well as sustaining the senescence of the cell [113,116]. At the early stages of tumorigenesis, CXCL1 inhibits tumor formation by enhancing senescence [113,119]. This means that there are two main factors in a senescent cell: p53, which inhibits cell division, and the CXCL1/CXCL8-CXCR2 axis, which is responsible for maintaining senescence as well as stimulating cell division and anchorage-independent growth when there is no active p53 [116]. If there is a mutation in the *TP53* gene, then the CXCL1/CXCL8-CXCR2 axis causes the proliferation of the cell, which may lead to tumor formation (Figure 2).

In tumors, senescence is involved in tumorigenesis. CXCL1 from a tumor cell induces the senescence of stromal fibroblasts [120] through the activation of p53 and p16 [120,121]. This mechanism has been observed in many cancers, such as colon cancer [120] and ovarian cancer [121], and leads to an increase in the expression and secretion of CXCL1 as well as other factors from the senescent fibroblast [121]. In other words, the cell exhibits SASP.

Under physiological conditions, senescence causes the activation of the immune system and the destruction of senescent cells [117]. However, in the tumor microenvironment, processes known as tumor immune evasion [122] inhibit the activation of the immune system. For this reason, SASP does not have an anti-tumor effect and promotes tumor growth, as it contains factors that increase proliferation and tumor cell migration as well as cause angiogenesis [115,116].

### 4.3. Cancer Stem Cells and Stemness

Cancer cells in a tumor are not a homogeneous population. There is a small population of cancer cells in the tumor called cancer stem cells, which are slow-cycling cells that show increased expression of DNA repair enzymes and transporters that remove xenobiotics, such as anticancer drugs outside their cell [123,124]. For this reason, these cells show resistance to chemotherapeutics and radiation therapy. After anticancer treatment, cancer stem cells reconstitute the cancer tumor; CXCL1 plays an important role in the physiology of these cells.

CXCL1 causes proliferation and self-renewal of cancer stem cells, as has been found in chronic myeloid leukemia (CML) [125], breast cancer [126,127], and glioblastoma [128]. CXCL1 also increases the stemness of cancer stem cells. Particularly, this chemokine increases the expression of CD133 in these cells, as shown in colorectal cancer [129]. Studies in mouse models have also shown that CXCR2 ligands induce quiescence and inhibit differentiation of premalignant hepatic cells [124]—an effect that is associated with the mechanistic target of rapamycin complex 1 (mTORC1) activation.

In cancer tumors, cancer stem cells may exhibit higher CXCL1 expression than cancer non-stem cells, as confirmed by a study on thyroid cancer [130]. CXCL1 expression in cancer stem cells is increased by various factors including neurotensin (NT) in hepatocellular carcinoma cancer stem cells [131] or insulin-like growth factor 1 (IGF-1) in lung cancer stem cells [132]. In the case of NT, increased CXCL1 expression is due to the activation of the RAF-1-ERK MAPK pathway [131]; this factor also increases CXCL8/IL-8 expression.

Cancer stem cells express CXCL1 [127] and CXCR2 [127]. Due to this, CXCL1 acts on these cells in an autocrine manner, increasing their numbers. This tumorigenic mechanism is significant in the development of breast cancer [127]. Importantly, the described effects of CXCL1 may depend on the type of cancer. For example, in thyroid cancer stem cells, CXCL8/IL-8 is a more important chemokine than CXCL1, due to the higher expression of CXCR1 than CXCR2 on cancer stem cells [130]. Similarly, in hepatocellular carcinoma cancer stem cells, the essential ligand of CXCR2 is not CXCL1 but CXCL8/IL-8 [131].

### 4.4. Angiogenesis and Lymphangiogenesis

In the early days of CXCL1 research, this chemokine was classified as a pro-angiogenic CXC chemokine with an ELR motif. Historically, the CXC chemokine sub-family was divided into ELR^+^ and ELR^−^ CXC chemokines [133]. ELR^+^ CXC chemokines had pro-angiogenic properties, in contrast to ELR^−^ CXC chemokines. In subsequent years, the ELR motif was shown to be important in the binding of ELR^+^ CXC chemokines to CXCR2 [134]. In other words, ELR^+^ CXC chemokines were ligands of CXCR2 [135] while ELR^−^ CXC chemokines were CXCR3 ligands [136]. The CXCR3 receptor is found in the S, G_2_, and M phases of the cell cycle in endothelial cells. Activation of this receptor blocks the proliferation of these cells, which means that CXCR3 ligands have anti-angiogenic properties. After the discovery of CXCL12, which lacks the ELR motif but has pro-angiogenic properties [137], the division of CXC chemokines into ELR^+^ and ELR^−^ became obsolete.

**Figure 3:**
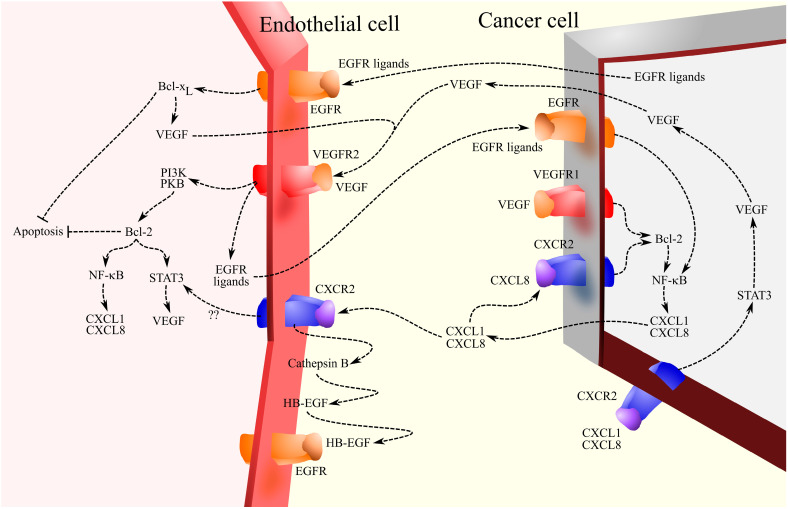
Interaction between endothelial cells and cancer cells via CXCL1, VEGF, and EGFR ligands. Activation of VEGFR1 and CXCR2 in cancer cells enhances Bcl-2 expression and activates NF-κB, leading to increased production of CXCL1 and CXCL8, which accumulate in the tumor microenvironment. Concurrently, EGFR activation in cancer cells further stimulates CXCL1 and CXCL8 expression through NF-κB signaling. Additionally, CXCR2 activation in cancer cells promotes VEGF production via STAT3 activation, reinforcing a pro-tumorigenic environment. Cancer cells also secrete EGFR ligands, which activate EGFR in endothelial cells, increasing Bcl-x_L_ expression. This in turn enhances VEGF production, which autocrinely activates VEGFR2 in endothelial cells. VEGFR2 activation upregulates Bcl-2, further promoting VEGF expression via STAT3 and increasing CXCL1 and CXCL8 levels through NF-κB signaling. This feedback loop may also stimulate the expression of EGFR ligands, amplifying the pro-tumorigenic signaling cascade. Additionally, CXCL1 and CXCL8 produced by cancer cells activate CXCR2 in endothelial cells, leading to EGFR transactivation and increased VEGF expression, further supporting tumor angiogenesis and progression.

The pro-angiogenic properties of CXCL1 are associated with the activation of CXCR2 on endothelial cells, as shown by experiments on bovine adrenal gland capillary endothelial cells [133], human umbilical vein endothelial cells (HUVECs) [138,139], and human dermal microvascular endothelial cells [138,139]. CXCL1 induces the migration, proliferation, and tube formation of endothelial cells. Also, in vivo experiments have confirmed the pro-angiogenic properties of CXCL1 [133,140]. CXCL1 induces angiogenesis in tumorigenesis because, in tumorigenesis, endothelial cells show CXCR2 expression (Figure 3) [141,142].

In tumor angiogenesis, CXCL1 interacts with vascular endothelial growth factor receptor (VEGFR) and EGFR ligands, as all these factors increase each other’s expression and thus act cooperatively. Vascular endothelial growth factor (VEGF) via VEGFR2-phosphatidylinositol-4,5-bisphosphate 3-kinase (PI3K)-PKB/Akt increases Bcl-2 expression in microvascular endothelial cells [143,144,145]. Also, VEGF can activate VEGFR1 on tumor cells, increasing Bcl-2 expression, as shown by experiments on head and neck squamous cell carcinomas [146]. Bcl-2 expression in cancer cells, such as in prostate cancer cells, may depend on CXCR2 [147].

As Bcl-2 is an anti-apoptotic protein [143], VEGF increases the viability of endothelial cells, which is an important mechanism for promoting angiogenesis. In addition, Bcl-2 can increase VEGF expression via signal transducer and activator of transcription (STAT)3 in microvascular endothelial cells [145]. Bcl-2 causes an increase in the inhibitory activity of NF-κB kinase (IKK) and thus an increase in NF-κB activation [144], which leads to an increase in CXCL1 and CXCL8/IL-8 expression in microvascular endothelial cells [144,145,148] and in cancer cells [146]; this means that VEGF increases CXCL1 expression by increasing Bcl-2 expression. This cascade can be initiated by epidermal growth factor (EGF) [148], which increases the expression of Bcl-x_L_ in endothelial cells, as shown by experiments on human dermal microvascular endothelial cells [148]. Subsequently, Bcl-x_L_ increases the expression of VEGF, which increases the expression of Bcl-2 and then the expression of CXCL1 and CXCL8/IL-8.

VEGF can also increase the expression of EGFR ligands, which increases the expression of CXCL1. The interaction of VEGF, EGFR, and CXCL1 may be a part of intercellular communication in cancer. VEGF from tumor cells may increase the expression of EGFR ligands in endothelial cells via VEGFR2 [149], which is followed by the secretion of EGFR ligands by endothelial cells. Activation of EGFR on cancer cells results in the activation of NF-κB and increased expression of CXCL1 and CXCL8/IL-8, which leads to the recruitment of endothelial cells [149,150].

CXCL1 can also influence the expression of VEGF and the activation of EGFR. CXCL1 increases VEGF-A expression, as shown by experiments on HUVECs [139] and studies of melanoma tumors with CXCL1 silenced [151]. In gastric cancer cells, CXCR2 activation results in STAT3 activation, which increases VEGF expression [99]. CXCL1 can also cause transactivation of the EGFR. CXCR2 activation causes cathepsin B activation in microvascular endothelial cells [152], which leads to the release of HB-EGF, an EGFR ligand. Experiments on HUVECs and human dermal microvascular endothelial cells have shown that CXCL1 increases EGF expression [138]. EGFR ligands activate their receptor, thereby causing endothelial cells to migrate to areas with an elevated level of CXCL1. CXCL1 acts not only on endothelial cells but also on endothelial progenitor cells (EPCs). It causes the transmigration of these cells and thus the recruitment of EPCs into the forming blood vessels in the tumor [153,154,155]. Also, CXCL1 induces EPC proliferation, differentiation, and the formation of capillary-like structures. CXCL1, similarly to other CXCR2 ligands, can also induce angiogenesis by acting on CXCR2^+^ cancer stem cells [156]. This is particularly relevant in glioblastoma multiforme tumors, where these cells exhibit vascular mimicry.

CXCL1 can also indirectly cause angiogenesis. It causes the recruitment of neutrophils into the tumor niche, which are then transformed into tumor-associated neutrophils (TANs) [157]. While TANs secrete VEGF [158], the main pro-angiogenic mechanism of these cells is an increase in VEGF bioavailability, enabled by MMP9, secreted by TANs. In the tumor niche, TANs are a significant source of MMP9 [159,160,161]. This metalloproteinase does not cleave VEGF165 but causes the digestion of heparan sulfates, which leads to the release of this growth factor from the ECM [162]. However, MMP9 can also cause angiogenesis independently of VEGF [163], which is likely related to the digestion of ECM by MMP9 and the release of other pro-angiogenic factors [164]. TANs also secrete oncostatin M [165], which can induce the secretion of VEGF from tumor cells, as shown in breast cancer cells. Another factor with pro-angiogenic properties that is secreted by TANs is Bv8/prokineticin 2 [166].

CXCL1 also participates in lymphangiogenesis and lymph node metastasis. Factors secreted by cancer cells induce an increase in the expression and secretion of CXCL1 by lymphatic endothelial cells (LECs) [167,168,169]. The CXCL1 secreted by LECs and other cells in the tumor niche act on LECs, which is followed by tube formation of LECs and lymphangiogenesis [168]. In addition, LECs migrate to areas with high CXCL1 levels [168]. CXCL1 also causes tumor cells to migrate into lymphatic blood vessels, which leads to lymph node metastasis. This mechanism has been demonstrated in breast cancer [169], gastric cancer [168,170], and oral tongue squamous cell carcinoma [167]. Nevertheless, this effect may depend on the type of cancer; for example, in prostate cancer, CCL7 is a more significant chemokine in this process than CXCL1 [169].

### 4.5. Cancer Immune Evasion

In cancer tumors, a very significant tumor mechanism is cancer immune evasion. This process involves inhibition of the anti-tumor immune response by various mechanisms that occur in the tumor microenvironment [122]. One of these components is CXCL1. This chemokine induces the recruitment of MDSCs into the tumor niche, which are cells with immunosuppressive properties [43,65,171]. CXCL1 also decreases the expression of human leukocyte antigen-group DR (HLA-DR) and IL-12p70 in activated dendritic cells [172], which inhibits the function of these cells in the anti-tumor response. On the other hand, CXCL1 does not affect the expression of IL-1β, IL-10, and TNF-α in these cells [172]. CXCL1 also increases programmed death-ligand 1 (PD-L1) expression in tumor cells as shown by experiments on glioblastoma [101]. PD-L1 is a trans-membrane protein that causes anergy of anti-tumor lymphocytes and thus protects cancer cells against the immune system [122].

### 4.6. Effects on Tumor-Associated Cells

#### 4.6.1. Adipocytes

Various cells, such as adipocytes, are recruited into the cancer tumor and transformed by the tumor microenvironment into cancer-associated adipocytes [173]. The significance of these cells is best understood in breast cancer, where cancer-associated adipocytes in the tumor niche secrete leukemia inhibitory factor (LIF) and thereby increase CXCL1 expression in cancer cells [174]. LIF activates STAT3 in cancer cells, which leads to an increase in CXCL1 expression in these cells. In turn, CXCL1 increases LIF expression in cancer-associated adipocytes. This means that there is a feedback loop between breast cancer cells and cancer-associated adipocytes—a mechanism involving CXCL1.

#### 4.6.2. Cancer-Associated Fibroblasts

Fibroblasts may be important in cancer formation. In old age, these cells secrete increased amounts of CXCR2 ligands, including CXCL1 [175]. This process has been noted in the prostate, which has been associated with benign prostatic hypertrophy, a condition preceding prostate cancer.

In the tumor niche, CAFs are the fibroblasts recruited and transformed by the tumor microenvironment. These cells can be distinguished into myofibroblastic CAFs and inflammatory CAFs [176,177]. Myofibroblastic CAFs are characterized by the high expression of α smooth muscle actin (αSMA) and produce ECM components. Inflammatory CAFs show high expression and secretion of pro-tumorigenic secreted factors including CXCR2 ligands (CXCL1, CXCL6/GCP-2, CXCL8/IL-8), CC motif chemokine ligand (CCL)5/regulated on activation, normally T cell expressed and secreted (RANTES), VEGF, granulocyte–macrophage colony-stimulating factor (GM-CSF), IL-6 and PGE_2_ produced by cyclooxygenase-2 (COX-2) [178,179]. This secretory phenotype is associated with NF-κB activation in inflammatory CAFs [179]. Exposure of CAFs to CXCR2 ligands, including CXCL1, leads to the enhancement of their secretory phenotype, i.e., their change into inflammatory CAFs [176].

CAFs in a tumor can arise from fibroblasts that are present in the parent tissue. CAFs can also be recruited from cells that are in other tissues and then transformed into CAFs. An example of this is white adipose tissue (WAT) adipose stromal cells (ASCs) [180]. Under the influence of CXCL1 as well as other CXCR2 ligands, ASCs are mobilized from WAT and then recruited to the tumor niche, as demonstrated in obese prostate cancer patients. ASCs are then transformed into myofibroblasts by CXCL1. CAFs can also arise from recruited bone marrow MSCs that differentiate into myofibroblasts [181].

In cancer tumors, in addition to cancer cells, CAFs also secrete CXCL1 into the tumor microenvironment. This has been demonstrated in cancers such as bladder cancer [182], colorectal cancer [14], malignant melanoma [183], and pancreatic ductal adenocarcinoma [184]. In prostate cancer, CXCL1 expression occurs in stromal myofibroblasts [185], and in colorectal cancer, the main source of CXCL1 is myofibroblasts [14]. In esophageal squamous cell carcinoma [186] and oral squamous cell carcinoma [187], CAFs are the most important sources of CXCL1. Importantly, CAFs must interact with cancer cells to produce large amounts of CXCL1, as shown by experiments on oral squamous cell carcinoma [187].

CXCL1 expression in CAFs is upregulated by cancer cells [188]. Cancer cells secrete extracellular vesicles—a type of intercellular communication in which various miRNA, mRNA, and proteins are transferred between cells. One of the elements carried by extracellular vesicles from the cancer cell are miRNA such as miR-155, miR-193b and miR-210 [189], which cause an increase in CXCL1 expression in fibroblasts in the tumor niche, such as in gastric cancer. Heat shock protein 90 (HSP90) and phosphorylated IKKα/β are also found in extracellular vesicles secreted from cancer cells under hypoxia [183]. The IKKα/β complex of HSP90 is introduced into CAFs, which activates NF-κB in these cells. This results in the increased expression of genes dependent on this transcription factor, including CXCL1 [183]. Extracellular vesicles secreted by cancer cells can also directly increase CXCL1 expression by transferring CXCL1 mRNA between cells [190].

Other factors also increase CXCL1 expression in CAFs. An example of this is IL-1α from a tumor cell, which increases CXCL1 expression, as well as enhancing the secretory phenotype and decreasing the myofibroblast phenotype of CAFs [36,191]. Another factor that increases CXCL1 expression in CAFs is tumor changes in ECM. Myofibroblasts cause the unfolding of the type III domains of fibronectin [87]. Subsequently, such modified fibronectin increases CXCL1 expression in CAFs, particularly in lung squamous cell carcinoma tumors [87]. Another ECM in tumors that increases CXCL1 expression in CAFs is laminin subunit gamma 1 (LAMC1) [192].

Also, the loss of responsiveness to TGF-β through the loss of TGF-β type II receptor (TβRII) expression may be responsible for the increase in CXCL1 expression in CAFs [71]. This process frequently occurs in many cancers, such as in prostate cancer [71]. TGF-β is a downregulator of CXCL1 expression. For this reason, cells that have lost responsiveness to this cytokine show increased expression of CXCL1 and CXCL16 [71]. This process is significant because the CXCL1 and CXCL16 secreted by CAFs increase the adhesion capacity of tumor cells to type I collagen, the main component of ECM in bones. Thus, this process facilitates the formation of bone metastasis.

CXCL1 produced by CAFs may contribute to resistance to anticancer treatment. The expression of this chemokine in CAFs is increased by radiotherapy, which is a mechanism of radioresistance, at least in esophageal squamous cell carcinoma [186].

In a tumor, CXCL1 acts on CAFs and causes a decrease in fibulin-1 expression [193]. This is induced by the activation of NF-κB, which forms a complex with histone deacetylase 1 (HDAC1) that reduces the acetylation of histones at the NF-κB-binding site in the fibulin-1 promoter [8]. This mechanism has been observed in prostate cancer cells, where CXCL1 decreased the expression of fibulin-1 [8], a protein that is involved in ECM function. This process is important in the development of many cancers, including colorectal cancer and prostate cancer. CXCL1 also induces the expression of connective tissue growth factor (CTGF) in CAFs [194]. This growth factor is important in the development of tumors, particularly in pancreatic ductal adenocarcinoma.

Factors secreted from the tumor cells, including CXCL1, cause the senescence of stromal fibroblasts [120]. In this state, these cells exhibit a SASP [115,116], dependent on p53 in the fibroblast [120,121]. A cancer cell often has a dysfunction in the *TP53* gene [29], causing it to avoid senescence. Senescent fibroblasts secrete various factors, including CXCL1, into the tumor microenvironment [120], although this process is not universal to all tumor types or all cases of a given cancer. Studies on various melanoma lines have shown that some lines increase CXCL1 expression in CAFs, while some have no effect on the expression of this chemokine [195].

#### 4.6.3. Mast Cells

Other cells in the tumor niche, although very rarely studied, are mast cells. These cells secrete CXCL1 in cancerous tumors, as confirmed by studies on the models of thyroid cancer [196] and adrenocortical carcinomas [197]. At the same time, the cancer processes in which the CXCL1 produced by these cells participates have been poorly studied.

#### 4.6.4. Myeloid-Derived Suppressor Cells

MDSCs are cells that reduce the immune system’s overly potent response [198]. In tumors, there is an accumulation of these cells, which has the effect of preventing the immune system from fighting the tumor. These cells can be divided into two main groups: granulocytic-myeloid-derived suppressor cells (G-MDSCs) and monocytic-myeloid-derived suppressor cells (M-MDSCs).

##### Granulocytic-Myeloid-Derived Suppressor Cells

CXCL1 induces the recruitment of G-MDSCs into the tumor niche, as these cells have CXCR2 expression [43,65,171,199]. At the same time, CXCL1 may be just one piece of a larger meshwork of associations between various factors in the tumor microenvironment.

A lot of cancers are pro-inflammatory in nature. For this reason, such cancers have increased levels of IL-1β, a cytokine that increases the expression of many factors that affect G-MDSC function [43]. Examples include granulocyte colony-stimulating factor (G-CSF) and Bv8/prokineticin 2, which are responsible for the mobilization from the bone marrow of G-MDSCs, but not for the recruitment of these cells into the tumor niche [43]. Also, IL-1β causes an increase in the expression of CXCR2 ligands, including CXCL1, chemokines that are responsible for the recruitment of G-MDSCs into the tumor niche [43,65,171]. Importantly, CXCL1 has no direct effect on the expansion of G-MDSCs in the bone marrow [200]. G-MDSCs are responsible for cancer immune evasion. They secrete S100A8/A9, which reduces the anti-tumor properties of CD8^+^ T cells [171]. MDSCs also secrete chemokines, including CXCR2 ligands [201], the expression of which is increased under hypoxia. MDSCs are also significant in tumorigenic processes beyond the primary tumor. Cancerous tumors secrete TGF-β into the bloodstream [202]. This results in increased expression of CXCR2 ligands in the liver. Also, CXCL1 from the primary tumor in colorectal cancer particularly translocates to the liver. CXCL1 in the liver causes infiltration of this organ by MDSCs [77]. MDSCs in the liver shape the pre-metastatic niche. This facilitates the formation of metastasis in the liver by colorectal cancer [77].

MDSCs not only cause cancer immune evasion in cancer tumors but also cause systemic immune downregulation caused by the cancer. Following the recruitment of these cells to the liver, they reduce the immune function of this organ [77], which leads to an overall reduction in immunity.

##### Monocytic-Myeloid-Derived Suppressor Cells

CXCL1 does not directly recruit M-MDSCs to the tumor niche because these cells do not exhibit high CXCR2 expression [65,200]. At the same time, once M-MDSCs are recruited into the tumor niche, induction of CXCR2 expression occurs in them [43]. CXCL1 can indirectly affect the recruitment of M-MDSCs to the tumor niche. Cancer causes chronic elevation of CXCL1 levels in the blood, which results in the expansion of M-MDSCs in the bone marrow [200,203]. At the same time, CXCL1 has no direct effect on the expansion of G-MDSCs [200]—the increase in M-MDSCs in the bone marrow occurs because of CXCR2 activation on granulocyte and macrophage progenitor cells (GMPs) [203], which reduces Sin3A-associated protein 18 (SAP18) expression in these cells. SAP18 binds to regulatory sequences at the *HRAS* and *PIK3CG* genes, leading to the decreased expression of the H-RAS proto-oncogene and PI3Kγ. This means that CXCL1 increases the expression of H-RAS and PI3Kγ in GMPs, and thus results in the increased activity of the ERK MAPK-STAT3 pathway. This leads to the differentiation of GMPs into M-MDSCs. The increased number of M-MDSCs in the bone marrow leads to an increase in the number of these cells in the blood and then to an increase in the recruitment of M-MDSCs into the tumor niche by other factors [200,203].

#### 4.6.5. Mesenchymal Stem Cells

CXCL1 induces the recruitment of bone marrow-derived mesenchymal stem cells (BM-MSCs) into the tumor niche [181]. At the same time, the importance of CXCL1 in this process is questioned, as BM-MSCs lack CXCR2 expression and therefore should not respond to CXCL1 [204]. In the tumor niche, BM-MSCs differentiate into myofibroblasts, including under the influence of extracellular vesicles from cancer cells [181,205]. In tumorigenesis, MSCs secrete many cytokines—a study on umbilical cord MSCs has shown that recruited MSCs secrete CXCL1, CXCL8/IL-8, CCL2/monocyte chemoattractant protein 1 (MCP-1) and IL-6 [128]. At the same time, the secretion of these pro-tumorigenic secreted factors is increased by acidosis, as confirmed by experiments on bone marrow-MSC [86]. Acidosis is an important element of the microenvironment of many cancers [85], which involves a decrease in pH under the influence of cancer cell metabolism. Other factors that increase the expression of CXCR2 ligands in the cells described are TNF-α [206] and IL-1β [207]. Another factor that increases CXCL1 expression in bone marrow MSC are extracellular vesicles from tumor cells [205]. Also, anticancer drugs, including carboplatin, increase CXCL1 expression in MSCs [208]. CXCL1 has a pro-tumor effect, and for this reason, an increase in CXCL1 expression in MSCs is a mechanism for increasing resistance to chemotherapy.

#### 4.6.6. Natural Killer Cells

Natural killer (NK) cells are cytotoxic cells that destroy cancer cells [209]. The anti-tumor activity in the tumor microenvironment of NK cells can be downregulated or even completely blocked by many factors and mechanisms [122]. NK cells can then begin to cooperate with cancer cells and other tumor-associated cells and promote tumor growth. For example, lung cancer cells under hypoxia secrete extracellular vesicles containing miR-150 [210], which directly downregulates CD226 expression in NK cells and thus alters the phenotype of NK cells. Such NK cells express VEGF, MMP, CCL2/MCP-1, IL-6, and CXCR2 ligands including CXCL1 and CXCL8/IL-8 [210] and therefore are involved in tumorigenesis.

#### 4.6.7. Tumor-Associated Dendritic Cells

CXCL1 is also secreted by tumor-associated dendritic cells (TADCs) into the tumor microenvironment [129]. These are dendritic cells recruited to the tumor niche, which, under the influence of the tumor microenvironment, have lost their anti-tumor properties and acquired the property of promoting tumor growth. Under the influence of factors from cancer cells, these cells secrete CXCR2 ligands, including CXCL1, CXCL2, and CXCL3 [129]. This process has been noted, for example, in colorectal cancer, where these chemokines played a significant role in cancer stem cell function and caused an increase in parathyroid hormone like hormone (PTHLH) expression in cancer cells, which promoted the formation of bone metastasis.

#### 4.6.8. Tumor-Associated Macrophages

Tumor-associated macrophages (TAMs) are monocytes that, under the influence of factors from the tumor microenvironment, have been transformed into macrophages that participate in tumor growth [211,212]. They are recruited to the tumor niche by multiple factors. Although the most significant chemokine in this process is CCL2/MCP-1 [213,214], CXCL1 can also recruit monocytes into the tumor niche, but not all of them. CD14^+^ monocytes have CXCR2 expression and can respond to CXCL1 [215], but the level of CXCR2 expression in these cells is much lower than in neutrophils; therefore, monocytes are recruited less efficiently than neutrophils through this pathway [216]. In contrast, CD16^+^ monocytes lack expression of this receptor [215]. CXCL1 can also induce TAM recruitment indirectly by increasing the expression of CCL2/MCP-1 in cancer cells [207], one of the most important chemokines causing TAM recruitment to the tumor niche.

Following recruitment, monocytes are transformed in the tumor niche into M2d TAMs [212], characterized by pro- and anti-inflammatory markers. Since TAMs have CXCR2 expression [217], they can respond to CXCL1, which enhances M2 polarization of macrophages in the tumor niche [182,217,218]. For this reason, high CXCL1 expression increases the number of M2-polarized TAMs in tumor tumorigenesis, as demonstrated in the glioblastoma [218] and prostate cancer models [217]. M2 macrophages are macrophages with anti-inflammatory, pro-angiogenic, and immunosuppressive properties [212]. At the same time, macrophages with M2 polarity do not secrete CXCL1, unlike M1 macrophages [219]. In the tumor niche, TAMs have M2d polarity [212], i.e., they are characterized by both pro- and anti-inflammatory properties. Due to their pro-inflammatory characteristics, TAMs secrete CXCL1 in large amounts into the tumor microenvironment [182,220,221,222,223] and may be a major source of this chemokine in some types of cancer, such as gastric cancer [223]. Factors that increase the expression of this chemokine in TAMs are tumor cells, which are, for example, associated with the action of TNF-α [223] and VEGF [77].

#### 4.6.9. Tumor-Associated Neutrophils

TANs are neutrophils recruited to the tumor niche and transformed into procancer cells that cause tumor growth [224]. At the same time, there are anticancer neutrophils with N1 polarization and procancer neutrophils with N2 polarization [225]. CXCL1 is a chemotactic factor for neutrophils [216,226,227,228]—for this reason, CXCL1 causes the recruitment of neutrophils into the tumor niche [185,228,229,230,231]. At the same time, CXCL1 recruits low-density neutrophils better than normal-density neutrophils [232].

During neutrophil recruitment, CXCL1 inhibits apoptosis of these cells, which leads to neutrophil accumulation at sites of high CXCL1 expression in this tumor [233]. Under the influence of factors from the tumor microenvironment, neutrophils transform into TANs. At the same time, neutrophils show some plasticity in their phenotype. CXCL1 induces the transition of normal-density neutrophils towards a low-density state [232], indicating this important influence of CXCL1 not only in the recruitment but also in the differentiation and function of TANs in the tumor niche.

Another important aspect of tumor function is the balance of TAN and TAM recruitment [231]. A decrease in the recruitment of either group results in an increase in the recruitment of the other group. With a decrease in TAM recruitment, there is an increase in CXCR2 ligand expression, leading to an increase in TAN recruitment to the tumor niche. Conversely, a decrease in TAN recruitment results in an increase in the expression of colony-stimulating factor 1 (CSF1) and CCL2/MCP-1 in the tumor, leading to an increase in TAM recruitment.

Neutrophils in the bone marrow have high CXCR2 expression, but in TAN tumors, they have low CXCR2 expression [234]. This may be due to the transformation of these cells into procancer cells. At the same time, CXCR2 is important in TAN plasticity in the tumor niche. CXCR2 enhances the polarization of TANs into anti-tumor cells—for this reason, lowering CXCR2 expression on TANs increases the procancer polarization of these cells [235].

In tumorigenesis, TANs produce CXCL1 as well as other CXCR2 ligands [70,234]. CXCL1 is involved in tumorigenesis, and the CXCL1-CXCR2 axis is also important in tumor cell–neutrophil interactions. In the tumor niche, cancer cells produce CXCL1 [236], which leads to the chemotaxis of neutrophils into the vicinity of cancer cells, as neutrophils have CXCR2 expression. Cancer cells, particularly breast cancer cells, also have the expression of β2-integrins, while neutrophils have the expression of intracellular adhesion molecule-1 (ICAM-1) [236]. Both proteins can fuse with each other, which allows the two cells to interact directly. As a result, cancer cells begin to migrate [236]. TANs also participate in tumorigenesis through other pathways. These cells secrete lipocalin 2 (LCN2), which causes tumor cell migration through the activation of Src signaling and the induction of EMT [185]. TANs also cause angiogenesis, due to the secretion of CXCR2 ligands, which have pro-angiogenic properties [70,133,135,234]. TANs secrete VEGF [158]. TANs also secrete MMP9, which is responsible for VEGF bioavailability [70,159,160,161]. TANs secrete other proteases involved in tumor growth, including MMP2 [70]. TANs also secrete oncostatin M [165] and Bv8/prokineticin 2 [166], which have pro-angiogenic properties. Finally, TANs are involved in cancer immune evasion because they express arginase 1 (ARG1) and indoleamine 2,3-dioxygenase (IDO) [70].

#### 4.6.10. Regulatory T Cells

Regulatory T cell (T_reg_) are T cells that regulate an overly intense immune system response. At the same time, there is an accumulation of these cells in the cancer tumor, resulting in tumor immune evasion [237], i.e., a decrease in the anti-tumor immune system response in the cancer tumor. T_reg_ can be recruited to either the tumor niche or malignant pleural effusion depending on CXCL1 [74]. However, there is only one paper indicating the importance of CXCL1 in the recruitment of T_reg_. Probably, CXCL1 has a minor role in the recruitment of these cells and only in some models. Recruitment of T_reg_ to the tumor niche is accomplished through many pathways, such as CCL2 [238], CCR4 ligands (CCL17 and CCL22) [239], CCR7 ligands (CCL19 and CCL21) [240], CCL18 [241], CXCR3 ligands (PF4, CXCL9, CXCL10, CXCL11) [242], and CXCL12-CXCR4 axis [243]. CXCL1 may be one way to recruit these cells, but other secretory factors may be more important in this process.

T_reg_ cells can also arise from naive CD4^+^ T cells [244]. Naive CD4^+^ T cells are recruited to the tumor niche by CXCL1 due to the expression of CXCR2 on these cells. Under the influence of factors in the tumor microenvironment, they can differentiate into T_reg_, a process which involves CXCL1 [244]. CXCL1 induces the differentiation of these cells into T_reg_ and enhances the immunosuppressive function of these cells [244].

### 4.7. Migration and Metastasis of Cancer Cells

CXCL1 causes migration and invasion of cancer cells, which has been noted in many types of cancer, including bladder cancer [12], colorectal cancer [99,129,245], gastric cancer [16,101], glioblastoma [246] and prostate cancer [8,247]. It is associated with increased expression of MMP2, MMP7, MMP9 and MMP13 in cancer cells [12,129,246,248,249]. CXCL1 also induces cancer cell migration by inducing EMT of cancer cells [8,129]. Causing EMT of cancer cells is possible through the activation of Src family kinases [185]. This process is caused by CXCL1 as well as by other factors, including lipocalin 2 (LCN2), secreted by TANs recruited by CXCL1 into the tumor niche. Also important in the induction of EMT by CXCL1 is the activation of the CXCR2-PI3K-Snail pathway [96]. This causes a decrease in E-cadherin expression, resulting in the cancer cell acquiring a mesenchymal phenotype. CXCL1 also induces EMT through the activation of the NF-κB-sex-determining region Y-related (SRY) high-mobility group box 4 (SOX4) pathway, as observed in breast cancer cells [221].

Cancer cell migration can also occur through the cooperation of multiple types of chemokines. CXCR2 activation results in the activation of NF-κB and STAT3 [250], which leads to an increase in the expression of CXCR4 and CXCR2, respectively. This increases the action of CXCR2 ligands as well as CXCL12, leading to EMT and migration of cancer cells. This mechanism has been noted in gastric cancer [250]. During migration and invasion, tumor cells move from the developed cancerous tumor to the adjacent tissue, where they appear as either single cells or small clusters in non-cancerous tissue. This is called “tumor budding” [251], a process that is dependent on several factors. High expression of CXCR2 ligands, including CXCL1, is positively correlated with tumor budding, at least in colorectal cancer [252].

One of the “hallmarks of cancer” is metastasis [253]. It involves the colonization of distant organs by cancer cells from the primary tumor. At the same time, metastasis is a highly inefficient process. It is estimated that, from just 1 g of tumor tissue, about 1 million cancer cells are released into the blood in just one day [254]. These cells will produce only a single metastasis during the years of cancer.

The most likely site for metastasis to form and develop is the primary tumor. This means that circulating tumor cells return to the primary tumor [255]—a process called “tumor self-seeding”. Then, cancer cells recruited to the primary tumor proliferate in the primary tumor, which results in more aggressive cells with a greater capacity to resist apoptosis and an increased ability to migrate and metastasize to other organs. One of the factors driving the recruitment of circulating tumor cells into the tumor niche of the primary tumor is IL-6 and CXCR2 ligands, such as CXCL1 [255].

Due to the high inefficiency of metastasis formation, processes are required that facilitate this process, at least at one stage of metastasis. CXCL1 acts on some of these; this chemokine causes the release of more cancer cells into the blood. For this reason, blood levels of CXCL1 in patients with colorectal cancer [256] and breast cancer [256] are positively correlated with the number of circulating tumor cells. More tumor cells in the blood means a higher likelihood of metastasis.

CXCL1 also facilitates metastasis formation by affecting the formation of pre-metastatic niche [257]. A cancer cell will not become metastatic if it accidentally appears in a healthy tissue. For metastasis to form, a healthy tissue must be transformed into an environment that facilitates the growth of a cancerous tumor—a process called pre-metastatic niche formation. An example of this is the increase in CXCL1 expression in the liver in colorectal cancer [77], which results in the recruitment of MDSCs to the liver. Subsequently, MDSCs cause cancer cell survival in the liver, which means that MDSCs facilitate the formation of liver metastasis by colorectal cancer, whose primary tumor is in the intestines [77].

CXCL1 expression in a tumor cell is increased during EMT-induced cell migration. This is associated with the attachment of Snail to the CXCL1 promoter [46]. This directly causes an increase in the expression of CXCL1. Also, Snail increases the activation of NF-κB, which is the most important transcription factor that increases CXCL1 expression [46]. CXCL2 expression is also increased by these two mechanisms. Snail is an important transcription factor causing EMT in cancer cells [47]. For this reason, during EMT, a cancer cell increases the expression of CXCL1. Then, such a cell migrates within the organ or enters the blood. For this reason, circulating tumor cells show CXCL1 expression, which is important in further stages of invasion or metastasis.

CXCL1 is involved in the formation of bone metastasis. Cancer cells isolated from bone metastasis show a secretory phenotype that facilitates bone metastasis. At least in prostate cancer, cancer cells from bone metastasis show expression of fibroblast growth factor (FGF)3, FGF19, growth differentiation factor-15 (GDF-15), and CXCL1, among others [258]. GDF-15 can both cause osteoclast differentiation and inhibit osteoblast differentiation [259]. CXCL1 increases the adhesion capacity of tumor cells to type I collagen, a major component of ECM in bones, as observed in prostate cancer cells [71]. Due to this adhesion, circulating tumor cells are more easily retained in bones, which increases the likelihood of bone metastasis.

CXCL1 also causes osteolysis, which is related to the stimulation of osteoclast maturation by this chemokine [258,260,261,262]. Osteoclasts cause bone remodeling, which leads to the development of bone metastasis. Osteoclast precursor cells have CXCR2 expression [260,263]; following the differentiation of these cells into osteoclasts, CXCR2 expression is reduced [260]. CXCL1 also increases PTHLH expression in cancer cells [129]; PTHLH and parathyroid hormone (PTH) cause an increase in CXCR2 ligand expression in osteoblasts, as shown by studies in mouse and rat cells [263]. PTHLH, PTH, and CXCR2 ligands cause bone remodeling by acting on osteoclasts during bone metastasis formation.

In addition to cancer cells, CXCL1 in bones may also come from bone marrow adipocytes, which increase the expression of CXCR2 ligands under the influence of factors from cancer cells, as shown by experiments on mouse cells [261]. The number of bone marrow adipocytes is elevated in older people or those with obesity, meaning that if they have prostate cancer, they are more likely to have bone metastasis [261].

CXCL1 is also involved in the formation of lung metastasis. It is secreted by human pulmonary artery endothelial cells [264] and human pulmonary microvascular endothelial cells [265]. It acts on circulating tumor cells that express CXCR2—activation of this receptor leads to activation of NF-κB in these cells. This process is dependent on the activation of the CXCR2–focal adhesion kinase (FAK)–PI3K-Akt/PKB-NF-κB pathway. As a result of NF-κB activation, there is an increase in vascular cell adhesion molecule-1 (VCAM-1) expression in circulating tumor cells [264,266], which facilitates the attachment of tumor cells to blood vessel walls, particularly to β1-integrin [265]. VCAM-1 is also involved in transendothelial migration of tumor cells in the lungs. These mechanisms facilitate lung metastasis, as observed in osteosarcoma [264].

CXCL1 may also be involved in the formation of brain metastasis. The formation of Breast cancer brain metastasis (BCBM) is dependent on the ligands CXCR2 and c-Met [267]. c-Met triggers the secretion of IL-1β from cancer cells, which then induces the activation of NF-κB in astrocytes. This leads to the secretion of hepatocyte growth factor (HGF) by these cells and activation on the cancer cell of c-Met, thus increasing the expression of IL-1β and CXCR2 ligands, namely, CXCL1 and CXCL8/IL-8 [267]. These ligands cause angiogenesis in the early stages of brain metastasis, i.e., they allow perivascular niche formation. Also, brain metastatic papillary thyroid carcinoma tumors have higher CXCL1 expression than either non-brain metastatic papillary thyroid carcinoma or primary brain tumors [268]. This indicates some role of CXCL1 in the formation or function of brain metastasis, at least in papillary thyroid carcinoma.

CXCL1 may also be important in metastasis to the intraperitoneal cavity as found in ovarian cancer in a mouse model [269]. CXCR2 ligands are synthesized in omental milky spots, adipose tissues, and blood vessels, as shown by experiments on animal material. These ligands are important in the formation of intraperitoneal cavity metastasis of ovarian cancer [269].

CXCL1 is an important driver of lymphatic metastasis. Cancer cells secrete factors that increase CXCL1 expression in LECs [167,249]. CXCL1 then causes cancer cells to migrate into the lymphatic vasculature. Also, other secretory factors such as CXCL5, CXCL6, CCL2, CCL7, and CCL20 are secreted by LECs in greater amounts [167]. These factors can also cause lymph node metastasis by the same mechanism. CXCL1 can cause lymphangiogenesis if it acts on LECs [168]. CXCL1 can cause tumor cell migration. An increase in CXCL1 levels in areas of lymphangiogenesis causes cancer cells to enter lymphatic vessels. CXCL1 is also important in downstream lymph node metastasis. Cancer tumors secrete CXCL1, which results in the recruitment of G-MDSCs to the lymph node at the early stages of metastasis [270]. These cells secrete TGF-β1, which is important in the formation of lymph node metastasis.

Once metastasis is initiated, the primary tumor can inhibit further metastatic development. One component of this process is CXCL1. In a subline of cancer cells with a highly metastatic potential, CXCL1 causes an increase in E-cadherin expression and a decrease in MMP2 expression [271]. This means that CXCL1 from the primary tumor inhibits EMT in cancer cells located in metastasis. This mechanism has been noted in malignant melanoma [271]. For this reason, surgical removal of primary tumor malignant melanoma is sometimes followed by a rapid development of metastasis.

## 5. Whole-Body Dysfunctions Associated with Cancer

Cancer is not a disease involving just a particular single organ, but cancer affects the entire body. Advanced long-term cancer is associated with many systemic symptoms and dysfunctions of organs not directly affected by the disease. This is due to chronic inflammation throughout the body, associated with the cancer tumor’s release of various factors, including CXCL1, into the bloodstream. Examples of tumor-associated dysfunction include cancer cachexia [272], cancer-associated immunodeficiency [202,273], neuroinflammatory-mediated affective-like behaviors [274], bone cancer pain [275,276] and dysfunction of the circulatory system and kidneys [277].

Cancer cachexia is a symptom of advanced cancer involving a decrease in body weight, particularly muscle mass. The condition is associated with chronic inflammation throughout the body [272] and exposure of the muscles to cytokines such as TNF-α, IL-1β, IL-6, and interferon-γ (IFN-γ). CXCL1 also plays a role in this process. Patients suffering from many types of cancer have elevated levels of CXCL1 in their blood, resulting in elevated exposure of their muscles [142,186,278]. In addition, factors secreted by a tumor increase CXCL1 expression in the muscles [279], which leads to the infiltration of skeletal muscles by neutrophils and M2 macrophages which then interfere with the differentiation of satellite cells—muscle stem cells responsible for muscle regeneration. The disruption of satellite cells reduces the ability to regenerate muscles, which is one of the elements of cancer-associated muscle wasting. At the same time, CXCL1 seems to have a destructive effect only when chronically acting on muscles. In a short-term action, it stimulates satellite cell proliferation and increases muscle regeneration and growth, i.e., it is a myokine [280].

Cancer is also associated with a systemic reduction in immunity, for example, an increased risk of respiratory infections among patients with advanced cancer [273]. One component of cancer-associated immunodeficiency is CXCL1. CXCR2 ligands cause the expansion of M-MDSCs [200,203], increasing the number of these cells in the bone marrow and spleen and thus in the bloodstream, resulting in a reduced immune response. A malignant tumor also increases the number of G-MDSCs in the bloodstream [65]. It secretes TGF-β [202], a cytokine that causes an increase in the expression of CXCR2 ligands in the liver, which results in the infiltration of this organ by G-MDSCs and a decrease in its immune function. Decreased immune function is associated with susceptibility to respiratory infection in patients with advanced cancer—a result of a chronic elevation of CXCL1 levels in the blood. Short-term increases in CXCL1 result in the mobilization of neutrophils from the bone marrow and the enhancement of the immune system [281].

Advanced cancer is also associated with impaired circulatory and renal function [277]. Cancer induces the activation of neutrophils, which then form neutrophil extracellular traps (NETs). In part, this is related to an increase in CXCL1 levels in the blood, as this chemokine can enhance neutrophil function and NET formation [282]. NETs form complexes with platelets; such complexes accumulate in the bloodstream and in the kidneys, damaging these organs. An increase in CXCL1 expression in the kidneys increases the infiltration of this organ by neutrophils, which then damages the organ [277].

Animal experiments have shown that cancer causes neuroinflammation-mediated affective-like behaviors [274], although it is unclear whether this is a result of cancer or increased awareness of the condition. Experiments on rodents have shown that a cancer tumor increases the level of CXCR2 ligands in the blood [274] and increases the expression of CXCR2 ligands in the hippocampus [274], although the latter may be the result of increased levels of IL-1β [283,284]. An increase in the expression of CXCR2 ligands is sufficient to give rise to affective-like behaviors [284,285].

Bone cancer or bone metastases are associated with bone cancer pain, which has been studied in laboratory animals. Cancerous tumors in bone secrete various factors that increase the expression of CXCR2 ligands in astrocytes in the spinal cord [275,286] and periaqueductal gray [276]. These chemokines activate their receptor CXCR2 on neurons, which causes bone cancer pain [275,276]. The increase in CXCR2 ligand expression in the spinal cord may be dependent on the activation of ephrin receptor B1 (EPHB1) by ephrinB in astrocytes and microglial cells [287], which causes an increase in TLR4 expression and the secretion of the cytokines IL-1β and TNF-α.

## 6. Patient Characteristics Versus Cancer and CXCL1

### 6.1. Obesity and Cancer

It is estimated that about 39% of the world’s population is overweight [288]. The largest percentage of overweight and obese populations is in North America and Europe, where about 60% of the population is overweight and about 25% is obese; these values have been steadily increasing for more than 30 years. The levels of obesity and overweight are also high and steadily increasing in other regions of the world. Abnormally high body weight is associated with the risk of contracting many diseases, including liver disease [289], diabetes [290], coronary heart disease [290], and many others. However, obesity is not associated with an increased risk of all cancers [290]. It may only increase the risk of certain cancers, such as esophageal adenocarcinoma, renal cancer and leukemia, and in women, postmenopausal breast cancer and endometrial cancer [291]. A higher body mass index (BMI) is associated with a reduced risk of lung cancer and, in women, premenopausal breast cancer [291]. Obesity also increases cancer aggressiveness. Prostate cancer patients [292] with obesity have a higher mortality rate compared with lean individuals with this cancer. Cancer in a patient with obesity is different from cancer in a slim patient in terms of the tumor microenvironment. CXCL1 may be responsible for the differences in cancer between patients with different BMIs.

Obesity is associated with chronic inflammation. It causes increased levels of CXCL1 in the blood of non-cancerous individuals [293] and increased expression of this chemokine in omental adipose tissue [294]. Also, obesity is associated with increased CXCL1 expression in the tumor of many cancers, such as breast cancer [295] and prostate cancer [180]. In slim prostate cancer patients, CXCL1 expression is at low levels [180]. Also, saturated fatty acids (SFAs), especially palmitate, can increase CXCR2 ligand secretion from hepatocellular carcinoma cells, as shown by experiments on H4IIE rat hepatoma cells [296]. Autocrine activation of CXCR2 on these cells inhibits apoptosis caused by a lipotoxic environment. CXCL1 also induces the migration and metastasis of tumor cells and causes the recruitment of various cells into the tumor niche. Particularly, it causes the recruitment of G-MDSCs and thus contributes to cancer immune evasion [295].

CXCL1 may also be important in the communication between the cancer cell and cancer-associated adipocytes (CAAs) [174]. Under the influence of the tumor microenvironment, normal adipocytes are transformed into CAAs. The significance of these cells in breast cancer is very well understood [173]. They produce many factors including CCL2, CCL5, VEGF, leptin, adiponectin, IL-6, and pro-inflammatory cytokines such as IL-1β and TNF-α [173]. In addition, CAAs secrete LIF, which causes migration and invasion of breast cancer cells [174]. At the same time, LIF also causes a STAT3-dependent increase due to breast cancer cell secretion of CXCR2 ligands such as CXCL1, CXCL2, CXCL3, and CXCL8/IL-8. In turn, these chemokines cause an increase in LIF production by CAAs, dependent on the activation of the ERK MAPK-NF-κB and ERK MAPK-STAT3 pathways [174]. CXCR2 activation on adipocytes also increases CCL2 and CCL6 expression [297], an effect observed in obese mice but not in lean mice. Factors secreted by adipocytes lead to the recruitment of TAMs into the tumor niche and proliferation of tumor cells, as shown by experiments on ovarian cancer mice. This contributes to the progression of this cancer.

Obesity and adipocytes may also contribute to bone metastasis. In obese individuals as in older people, there is an increase in bone marrow adiposity [261], which means that the number of adipocytes in the bone marrow increases. As adipocytes are the source of CXCR2 ligands, in the initial stages of bone metastasis, a greater amount of CXCR2 ligands results in increased osteoclast activity and thus bone remodeling. CXCL1 also causes the mobilization from WAT of ASCs and the recruitment of these cells to the tumor niche, as demonstrated in a prostate cancer model [180]. However, this process is also dependent on other chemokines, including CXCL8/IL-8. In addition, CXCR1 appears to be the main receptor responsible for the mobilization and recruitment of ASCs by CXCL1 and CXCL8/IL-8 [180]. ASCs secrete many factors in large numbers, including CXCL1, CXCL8/IL-8, CX_3_C motif chemokine ligand 1 (CX_3_CL1)/fractalkine, CCL2, and plasminogen activator inhibitor 1 (PAI1) [298]. These factors are involved in tumorigenesis—they cause angiogenesis, migration, and metastasis of tumor cells and recruit various cells into the tumor niche.

Adiponectin, a factor produced by adipose tissue, causes an increase in CXCL1 secretion from ovarian cancer cells [299] and colon cancer cells [120,300]. CXCL1 is involved in cancer processes, including either angiogenesis or stromal cell senescence. As obese individuals have reduced levels of adiponectin in the blood [301,302], obesity may be associated with a reduced aggressiveness of ovarian cancer and colon cancer. Nevertheless, this does not offset other procancer mechanisms associated with obesity.

Another adipokine is leptin whose levels are positively correlated with BMI [303,304]. Studies on mice have shown that leptin causes an increase in the expression of IL-6, IL-1β, and keratinocyte-derived chemokine (KC) in the colon [305]. KC is a CXC sub-family chemokine, a mouse paralog for human CXCL1, almost always equated with this chemokine. The pro-inflammatory effects of leptin on the colon may contribute to chronic colon inflammation and colon cancer [305]. On the other hand, studies on HT-29 colon cancer cells have shown that leptin does not increase CXCL1 expression in these cells [300]. It is likely that leptin does not increase CXCL1 expression in colon cancer tumors, although its effect in other cancers needs to be confirmed experimentally.

Obesity also causes an increase in visfetin (other names NAMPT and PBEF) in the blood of healthy people [306] and visfatin production in tumors as shown by experiments on gastric cancer mice [307]. Visfatin is also produced by many cancers including breast cancer [308], colorectal cancer [309], melanoma [310], and prostate cancer [311]. Visferin stimulates cancer cell growth, enables anchorage-independent growth, and invasion of cancer cells [311]. It also induces M2 polarization of macrophages in the tumor niche which results in increased production and secretion of CXCL1 by TAM [308]. This chemokine is involved in cancer processes, including angiogenesis [133], migration, and invasion of cancer cells [308].

### 6.2. Patient Age

Cancers are associated with the aging of people, which is a certain mechanism, or consequence, of aging in the body. There are chronic inflammatory reactions in the tissues of older people. An example of this is prostate fibroblasts, which in older people secrete increased amounts of CXCR2 ligands, such as CXCL1 [175]. This leads to benign prostatic hypertrophy, which is a pre-cancerous condition of prostate cancer.

A greater patient age affects tumorigenic processes in gastric cancer tumors, but this depends on the type of cancer. In gastric cancer tumors, elevated levels of CXCL1 are found in older patients than in younger patients [312]. This was also confirmed by studies of colon adenocarcinoma tumors [313,314]. Other studies, for example, on colorectal cancer, have shown an inverse relationship—in patients under 65 years of age, CXCL1 expression in the tumor was higher than in those over 65 [315]. In prostate cancer, on the other hand, the age of the patient had no effect the level of CXCL1 in the tumor [316]. Older people have a higher number of adipocytes in the bone marrow [261]. These cells under the influence of cancer cells produce CXCR2 ligands, as shown by experiments on mouse cells. For this reason, bone marrow adipocytes facilitate the formation of bone metastasis in elderly people.

Due to advances in the knowledge of tumor mechanisms in cancerous tumors, therapeutic approaches that target only one tumor mechanism are being developed. This is called molecular targeted cancer therapy [317]. Such therapeutic approaches have great potential because they can individually approach each patient. To better develop such therapy, it is necessary to better understand the impact of a patient’s age on tumor mechanisms in a cancerous tumor. Currently, this research aspect is very rarely studied.

### 6.3. Fertility

Higher fertility reduces the likelihood of ovarian cancer in women [318]. This is related to CXCL1 as indicated by animal experiments. For example, experiments in mice have shown that nulliparous animals have reduced levels of CXCR2 ligands in the blood [319]. In turn, higher fertility results in decreased expression of CXCR2 ligands in omental fat band and peritoneal serous fluid [320]. In the case of ovarian cancer formation, the level of CXCR2 ligands in the omental fat band in nulliparous animals is higher than in parous mice [320].

Changes in the expression of CXCR2 ligands as well as other genes affect the development of ovarian cancer, particularly the peritoneal cancer index. Increased expression of CXCL1 in the omental fat band promotes the formation of intraperitoneal cavity metastasis of ovarian cancer [269,320]. This means that women who have given birth to more children are less likely to develop ovarian cancer, and if they do, this cancer is less likely to give rise to intraperitoneal cavity metastasis.

## 7. Summary

**Figure 4:**
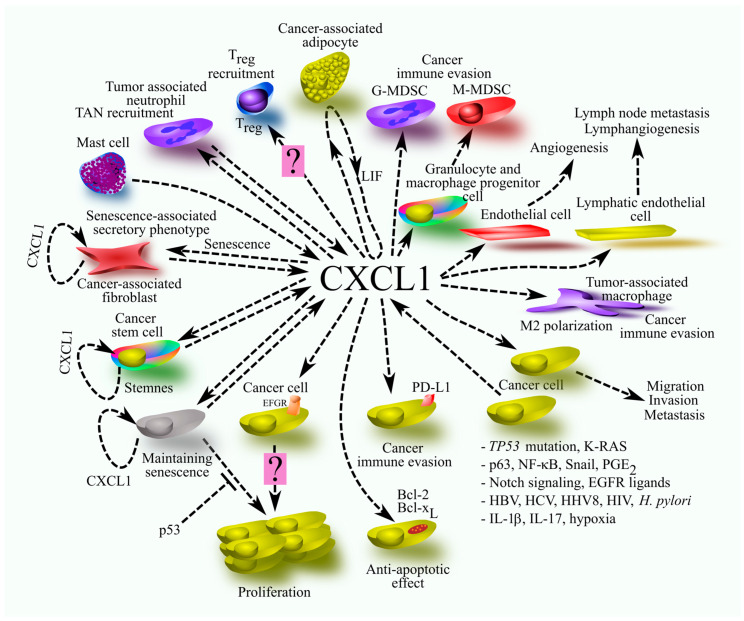
The involvement of CXCL1 in the cancer-related mechanisms discussed in this review. CXCL1 in the tumor microenvironment is produced by cancer cells as well as by certain tumor-associated cells, including CAFs, mast cells, and TANs. CXCL1 acts both on cancer cells and on tumor-associated cells.

CXCL1 participates in numerous cancer-related mechanisms that we summarize in the final figure of this article (Figure 4). Based on the available experimental studies, it can be stated that CXCL1 potentially influences almost all cancer-related processes, at least theoretically. However, it should be emphasized that each of them depends on many different factors (with CXCL1 representing only one of them), which vary in significance. In one type of cancer, a particular process may depend predominantly on one factor, whereas in another cancer, it may depend on a different factor, while the remaining ones may play only a minor role.

Additional complexity arises from the presence of other CXCR2 ligands. Besides CXCL1, six additional chemokines act as CXCR2 ligands and share very similar biological properties. The main differences between them lie in the regulation of their expression. As a result, some of the functions attributed here to CXCL1 may also be performed by these other chemokines.

In this review, we focus on the potential molecular mechanisms through which CXCL1 participates in cancer-related processes. At the same time, this article is primarily theoretical in nature. Its important limitation lies in the lack of analysis of the relative significance of CXCL1 in specific cancer-related processes across different tumor types. In other words, the involvement of CXCL1 in a particular cancer-related process may not occur in certain types of cancer.

The association between CXCL1 and cancer-related processes in specific cancers was previously analyzed using bioinformatic tools in our earlier article titled “Bioinformatic Analysis of the CXCR2 Ligands in Cancer Processes” (https://doi.org/10.3390/ijms241713287) [321]. In that study, we examined correlations in clinical samples not only for CXCL1 but for all CXCR2 ligands across the cancer-related processes discussed. For this reason, the present review can be considered a theoretical extension of that bioinformatic study. Both articles should therefore be read and interpreted together to obtain a more comprehensive understanding of the role of CXCL1 in cancer-related processes.

## Data Availability

No new data were created or analyzed in this study. Data sharing is not applicable to this article.

## Abbreviations

αSMA	α smooth muscle actin
ACKR1	Atypical chemokine receptor 1
ARG1	Arginase 1
ASC	Adipose stromal cell
BBP	Benzyl butyl phthalate
BMI	Body mass index
BM-MSC	Bone marrow-derived mesenchymal stem cell
CAA	Cancer-associated adipocyte
CAF	Cancer-associated fibroblast
CDK2	Cyclin-dependent kinase 2
CML	Chronic myeloid leukemia
COX-2	Cyclooxygenase-2
CSF1	Colony-stimulating factor 1
CTGF	Connective tissue growth factor
CX_3_CL1	CX_3_C motif chemokine ligand 1
CXCL	CXC motif chemokine ligand
CXCL4L1	CXC motif chemokine ligand 4 variant 1
CXCR	CXC motif chemokine receptor
DARC	Duffy antigen receptor for chemokines
DEHP	Di-(2-ethylhexyl) phthalate
ECM	Extracellular matrix
EGF	Epidermal growth factor
EGFR	Epidermal growth factor receptor
EMT	Epithelial-to-mesenchymal transition
EPC	Endothelial progenitor cell
EPHB1	Ephrin receptor B1
ERK	Extracellular signal-regulated kinase
FADD	Fas-associated via death domain
FAK	Focal adhesion kinase
FGF	Fibroblast growth factor
G-CSF	Granulocyte colony-stimulating factor
GDF-15	Growth differentiation factor 15
GLUT1	Glucose transporter type 1
GM-CSF	Granulocyte–macrophage colony-stimulating factor
G-MDSC	Granulocytic-myeloid-derived suppressor cell
GMP	Granulocyte and macrophage progenitor cell
GPR35	G-protein-coupled receptor 35
HB-EGF	Heparin-binding EGF-like growth factor
HBV	Hepatitis B virus
HCV	Hepatitis C virus
HDAC1	Histone deacetylase 1
HEYL	Hes-related family bHLH transcription factor with YRPW motif like
HGF	Hepatocyte growth factor
HHV8	Human herpes virus-8
HIV	Human immunodeficiency virus
HK2	Hexokinase 2
HLA-DR	Human leukocyte antigen-group DR
HSP90	Heat shock protein
HUVEC	Human umbilical vein endothelial cell
ICAM-1	Intracellular adhesion molecule 1
IDO	Indoleamine 2,3-dioxygenase
IFN-γ	Interferon-γ
IGF-1	Insulin-like growth factor 1
IKK	Inhibitor of NF-κB kinase
IL	Interleukin
KC	Keratinocyte-derived chemokine
KSHV	Kaposi’s sarcoma-associated herpes virus
LAMC1	Laminin subunit gamma 1
LCN2	Lipocalin 2
LDHA	Lactate dehydrogenase A
LEC	Lymphatic endothelial cell
LIF	Leukemia inhibitory factor
MAPK	Mitogen-activated protein kinase
MCP-1	Monocyte chemoattractant protein 1
Mdm2	Murine double minute 2
M-MDSC	Monocytic-myeloid-derived suppressor cell
MMP	Metalloproteinase
MSC	Mesenchymal stem cell
mTORC1	Mechanistic target of rapamycin complex 1
NET	Neutrophil extracellular trap
NF-κB	Nuclear factor κB
NIK	NF-κB-inducing kinase
NK	Natural killer cell
NT	Neurotensin
PAI1	Plasminogen activator inhibitor 1
PD-L1	Programmed death-ligand 1
PF4	Platelet-derived factor 4
PGE_2_	Prostaglandin E_2_
PI3K	Phosphatidylinositol-4,5-bisphosphate 3 kinase
PKB	Protein kinase B
PTH	Parathyroid hormone
PTHLH	Parathyroid hormone-like hormone
RANTES	Regulated on activation, normally T cell expressed and secreted
RIPK1	Receptor interacting serine/threonine kinase 1
SAP18	Sin3A associated protein 18
SASP	Senescence-associated secretory phenotype
SDF-1	Stromal-derived factor-1
SETD2	Histone H3 lysine 36 methyltransferase SET-domain-containing 2
SFA	Saturated fatty acid
SOX4	High-mobility group box 4
SRY	Sex-determining region Y-related
STAT	Signal transducer and activator of transcription
TADC	Tumor-associated dendritic cell
TAM	Tumor-associated macrophage
TAN	Tumor-associated neutrophil
TNF-α	Tumor necrosis factor-α
T_reg_	Regulatory T cell
TβRII	TGF-β type II receptor
VCAM-1	Vascular cell adhesion molecule 1
VEGFR	Vascular endothelial growth factor receptor
WAT	White adipose tissue
